# SFTSV nucleoprotein mediates DNA sensor cGAS degradation to suppress cGAS-dependent antiviral responses

**DOI:** 10.1128/spectrum.03796-23

**Published:** 2024-05-07

**Authors:** Ze-zheng Jiang, Min Chu, Li-na Yan, Wen-kang Zhang, Bang Li, Jiao Xu, Zhong-xin Zhao, Hui-Ju Han, Chuan-min Zhou, Xue-jie Yu

**Affiliations:** 1 State Key Laboratory of Virology, School of Public Health, Wuhan University, Wuhan, Hubei, China; 2 Reproductive Medicine Center, The Affiliated Yantai Yuhuangding Hospital of Qingdao University, Yantai, Shandong, China; 3 Department of Laboratory Medicine, Linyi People’s Hospital, Linyi, Shandong, China; 4 School of Public Health, Shandong First Medical University & Shandong Academy of Medical Sciences, Jinan, Shandong, China; Instituto de Histología y Embriología de Mendoza (IHEM), Mendoza, Argentina

**Keywords:** cGAS, innate immunity, nucleoprotein, SFTSV, RNA virus, autophagy

## Abstract

Cyclic GMP-AMP synthase (cGAS) is an important DNA pattern recognition receptor that senses double-stranded DNA derived from invading pathogens or self DNA in cytoplasm, leading to an antiviral interferon response. A tick-borne Bunyavirus, severe fever with thrombocytopenia syndrome virus (SFTSV), is an RNA virus that causes a severe emerging viral hemorrhagic fever in Asia with a high case fatality rate of up to 30%. However, it is unclear whether cGAS interacts with SFTSV infection. In this study, we found that SFTSV infection upregulated cGAS RNA transcription and protein expression, indicating that cGAS is an important innate immune response against SFTSV infection. The mechanism of cGAS recognizing SFTSV is by cGAS interacting with misplaced mitochondrial DNA in the cytoplasm. Depletion of mitochondrial DNA significantly inhibited cGAS activation under SFTSV infection. Strikingly, we found that SFTSV nucleoprotein (N) induced cGAS degradation in a dose-dependent manner. Mechanically, N interacted with the 161-382 domain of cGAS and linked the cGAS to LC3. The cGAS-N-LC3 trimer was targeted to N-induced autophagy, and the cGAS was degraded in autolysosome. Taken together, our study discovered a novel antagonistic mechanism of RNA viruses, SFTSV is able to suppress the cGAS-dependent antiviral innate immune responses through N-hijacking cGAS into N-induced autophagy. Our results indicated that SFTSV N is an important virulence factor of SFTSV in mediating host antiviral immune responses.

**IMPORTANCE:**

Severe fever with thrombocytopenia syndrome virus (SFTSV) is a tick-borne RNA virus that is widespread in East and Southeast Asian countries with a high fatality rate of up to 30%. Up to now, many cytoplasmic pattern recognition receptors, such as RIG-I, MDA5, and SAFA, have been reported to recognize SFTSV genomic RNA and trigger interferon-dependent antiviral responses. However, current knowledge is not clear whether SFTSV can be recognized by DNA sensor cyclic GMP-AMP synthase (cGAS). Our study demonstrated that cGAS could recognize SFTSV infection via ectopic mitochondrial DNA, and the activated cGAS-stimulator of interferon genes signaling pathway could significantly inhibit SFTSV replication. Importantly, we further uncovered a novel mechanism of SFTSV to inhibit innate immune responses by the degradation of cGAS. cGAS was degraded in N-induced autophagy. Collectively, this study illustrated a novel virulence factor of SFTSV to suppress innate immune responses through autophagy-dependent cGAS degradation.

## INTRODUCTION

Severe fever with thrombocytopenia syndrome virus (SFTSV) is an emerging tick-borne Bunyavirus, which was first reported in China in 2009 (1) and caused a severe hemorrhagic fever termed severe fever with thrombocytopenia syndrome (SFTS). Other manifestations of SFTS include anorexia, myalgia, chills, lymphadenopathy, lymphocytopenia, and multiple organ failure. SFTS has a high case fatality rate ranging from 6% to 30% (1–3). While tick bites are the main route of SFTSV transmission, SFTSV can also be transmitted to humans through mucosal membranes via body fluids or aerosols from person-to-person or animal-to-person contact (4–6). Up to now, SFTSV has been reported in East and Southeast Asian countries including China, South Korea, Japan, Vietnam, Thailand, and Myanmar (1, 7–10), and serological evidence of SFTSV was detected from the sera of the healthy population in Pakistan (11). Heartland virus, another tick-borne Bunyavirus reported in the United States in 2012, was phylogenetically associated with SFTSV (12), which suggested that tick-borne pathogenic Bunyavirus may have a wide global distribution. In view of the threat of SFTSV to human health, the World Health Organization has listed SFTSV on Blueprint Priority Diseases in 2018 (13).

SFTSV is a negative-sense single-stranded RNA virus, which contains a tri-segmented genome including large (L), medium (M), and small (S) segments. The L segment contains 6,368 nucleotides encoding the RNA-dependent RNA polymerase (RdRp), which is responsible for catalyzing viral genome replication and transcription. The M segment contains 3,378 nucleotides encoding the glycoprotein, which is responsible for mediating the attachment and fusion of virus and host cells. The S segment contains 1,744 nucleotides encoding nucleoprotein (N) and the nonstructural protein (NSs) using an ambisense coding strategy. The NSs is considered as the main SFTSV virulence factor (14, 15), which is considered to form inclusion bodies (IBs) to sequester multiple interferon-related proteins, such as RIG-I, TRIM25, TBK1, IKKε, IRF3, and IRF7, to suppress antiviral innate immune responses (16–22). Furthermore, the NSs also plays an important role in immunosuppression by promoting the production of the anti-inflammatory cytokine IL-10 (23).

Pattern recognition receptors (PRRs), which recognize pathogen-associated molecular patterns (PAMPs), are important mammalian defense mechanisms to resist pathogen invasion. As a single-stranded RNA virus, current investigation showed that SFTSV infection was recognized via canonical RNA sensors RIG-I and MDA5 and a novel RNA sensor SAFA. Briefly, upon recognition, RIG-I and MDA5 recruit mitochondrial antiviral signaling protein to activate TBK1 and induce type I interferon (IFN) production (18). A recent study indicated that SAFA could recognize SFTSV infection in a stimulator of interferon genes (STING)-dependent manner (24–26). However, it remains unclear whether additional nucleic acid PRRs are involved in SFTSV recognition. The cyclic GMP-AMP synthase (cGAS), a DNA sensor, recognizes double-stranded DNA derived from invading pathogens or self-DNA invading cytoplasm, leading to an antiviral interferon response. Typically, cytosolic cGAS predominantly localizes to the inner leaflet of the plasma membrane, which allows cGAS to detect invading pathogens in time (27). Upon binding to dsDNA, cGAS assembles into a dimer and catalyzes the production of cyclic GMP-AMP (cGAMP). cGAMP subsequently binds to a STING on the endoplasmic reticulum (ER) (28). Then, STING travels to the Golgi through the ER–Golgi intermediate compartment and recruits TBK1 to activate IRF3-dependent antiviral innate immune responses. In addition, a few recent studies have demonstrated that cGAS can also recognize some RNA virus infections (29–32). However, it remains unclear whether cGAS is involved in the recognition of SFTSV and the mechanism of the interaction between cGAS and SFTSV. In this study, we aim to investigate the underlying interaction between DNA sensor cGAS and RNA virus SFTSV.

## RESULTS

### SFTSV infection activates cGAS-STING signaling pathway

**Fig 1:**
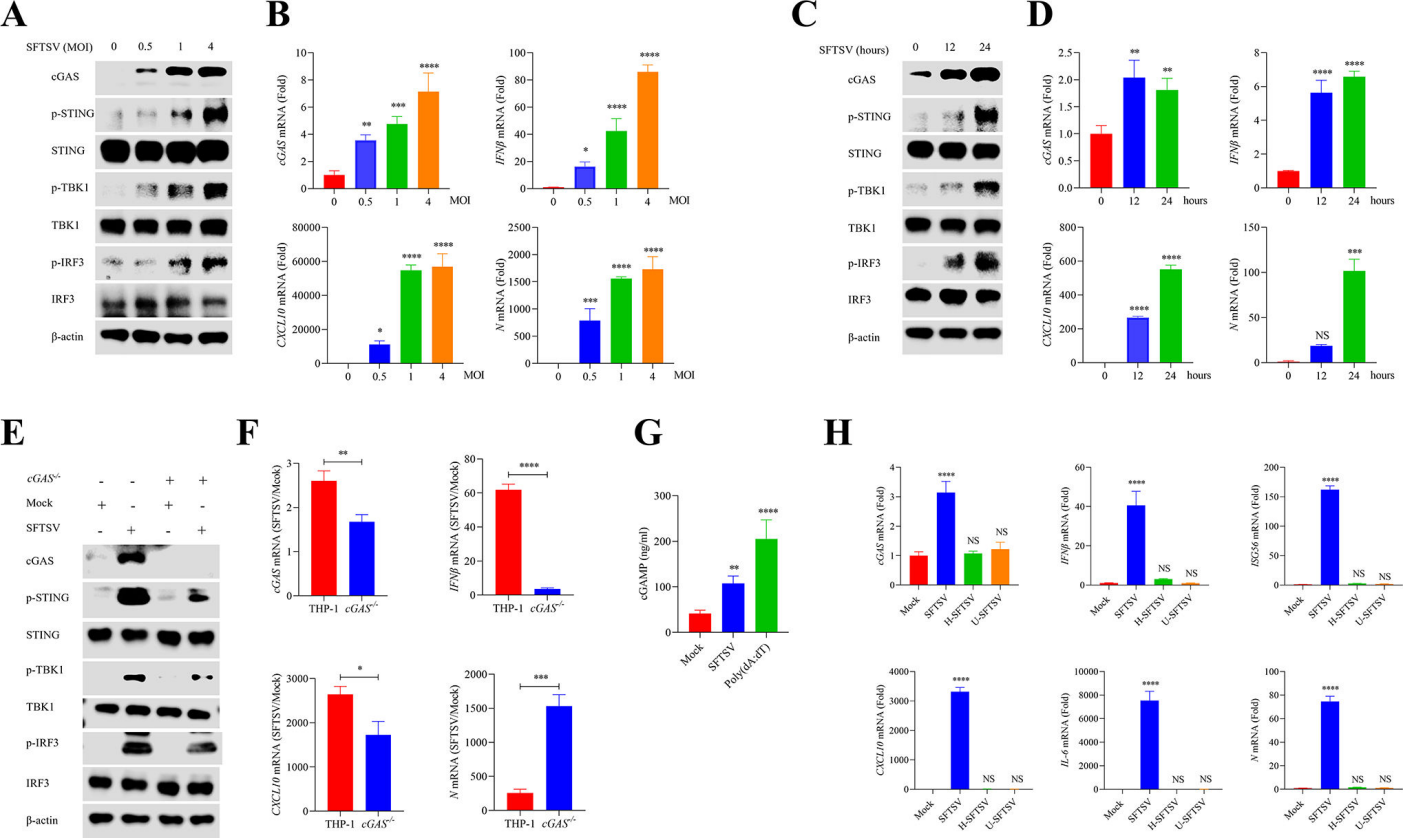
SFTSV infection activates the intracellular DNA sensor cGAS. (**A and B**) THP-1 cells were infected with SFTSV at the multiplicity of infection (MOI) of 0, 0.5, 1, and 4 for 24 h. cGAS-related proteins were determined with immunoblot analysis. The transcriptional levels of *cGAS*, *IFNβ*, *CXCL10*, and *N* were determined with RT-qPCR (one-way ANOVA). (**C and D**) THP-1 cells were infected with SFTSV at MOI of 0.5 for 0, 12, and 24 h. cGAS-related proteins were determined with immunoblot analysis. The transcriptional levels of *cGAS*, *IFNβ*, *CXCL10*, and *N* were determined with RT-qPCR (one-way ANOVA). (**E and F**) *cGAS* knockout and WT THP-1 cells were infected with SFTSV at MOI of 0 or 4 for 24 h. cGAS-related proteins were determined with immunoblot analysis. The transcriptional levels of *cGAS*, *IFNβ*, *CXCL10*, and *N* were determined with RT-qPCR; the fold increases of mRNA (SFTSV/Mock) were compared by Student’s *t*-test. (**G**) THP-1 cells were infected with SFTSV at MOI of 0.5 for 24 h or transfected with poly(dA:dT) (0.2 µg/µL) for 6 h. The cytosolic cGAMP was measured with enzyme-linked immunosorbent assay (ELISA) (one-way ANOVA). (**H**) THP-1 cells were infected with live SFTSV or heat-inactivated or ultraviolet-inactivated SFTSV at MOI of 0.5 for 24 h. The transcriptional levels of *cGAS*, *IFNβ*, *ISG56*, *CXCL10*, *IL-6*, and *N* were determined with RT-qPCR (one-way ANOVA).

THP-1 cells, a human leukemia monocytic cell line, were utilized to assess the status of the cGAS-STING signaling pathway under SFTSV infection. Immunoblot analysis showed that the protein level of cGAS was significantly increased and exhibited a dose-dependent manner under SFTSV infection (Fig. 1A), whereas the protein level of STING did not increase (Fig. 1A). In addition, we found that SFTSV infection promoted the phosphorylation of STING, TBK1, and IRF3 (Fig. 1A; Fig. S1A), which were the markers of activation of the cGAS-STING signaling pathway. Subsequently, we assessed the kinetics of the cGAS-STING signaling pathway by monitoring the transcriptional levels of cGAS-associated IFN and inflammatory genes. Reverse transcription quantitative real-time polymerase chain reaction (RT-qPCR) results showed that the transcriptional levels of *cGAS*, *IFNβ*, *ISG56*, *CXCL10*, and *TNFα* were elevated significantly in a dose-dependent manner during SFTSV infection (Fig. 1B; Fig. S1B). Similarly, immunoblot and RT-qPCR data showed that SFTSV infection activated the cGAS-STING signaling pathway in a time-dependent manner (Fig. 1C and D; Fig. S1C and D). These results suggested that cGAS could sense SFTSV to mediate an immune response against SFTSV infection. To further clarify the role of cGAS in SFTSV infection, the CRISPR/Cas9 technique (33) was utilized to knock out *cGAS* gene in THP-1 cells; the *cGAS* knockout THP-1 cells were termed as *cGAS^−/−^* THP-1. Interestingly, the *cGAS^−/−^* THP-1 cells showed marked reduction in the phosphorylation of STING, TBK1, and IRF3 under SFTSV infection (Fig. 1E; Fig. S1E), and the transcriptional levels of *cGAS*, *IFNβ*, *ISG56*, *CXCL10*, and *TNFα* were also significantly decreased in SFTSV-infected *cGAS^−/−^* THP-1 cells (Fig. 1F; Fig. S1F). Considering that the second messenger cGAMP is catalyzed by cGAS, cytosolic cGAMP was then detected in SFTSV-infected wild-type THP-1 cells. Poly(dA:dT), a synthetic analog of double-stranded DNA, was utilized as positive control to induce cGAS activation. The results showed that SFTSV infection effectively induced the production of cGAMP compared to uninfected THP-1 cells, albeit at a slightly lower level than poly(dA:dT) (Fig. 1G). In addition, we also observed that heat-inactivated (H-SFTSV) or ultraviolet-inactivated (U-SFTSV) SFTSV could not induce the activation of the cGAS-STING signaling pathway (Fig. 1H). Collectively, these data indicate that the cGAS-STING signaling pathway is tightly involved in recognizing RNA virus SFTSV infection.

### The impact of cGAS deficiency for host to suppress SFTSV replication *in vivo* and *in vitro*

**Fig 2:**
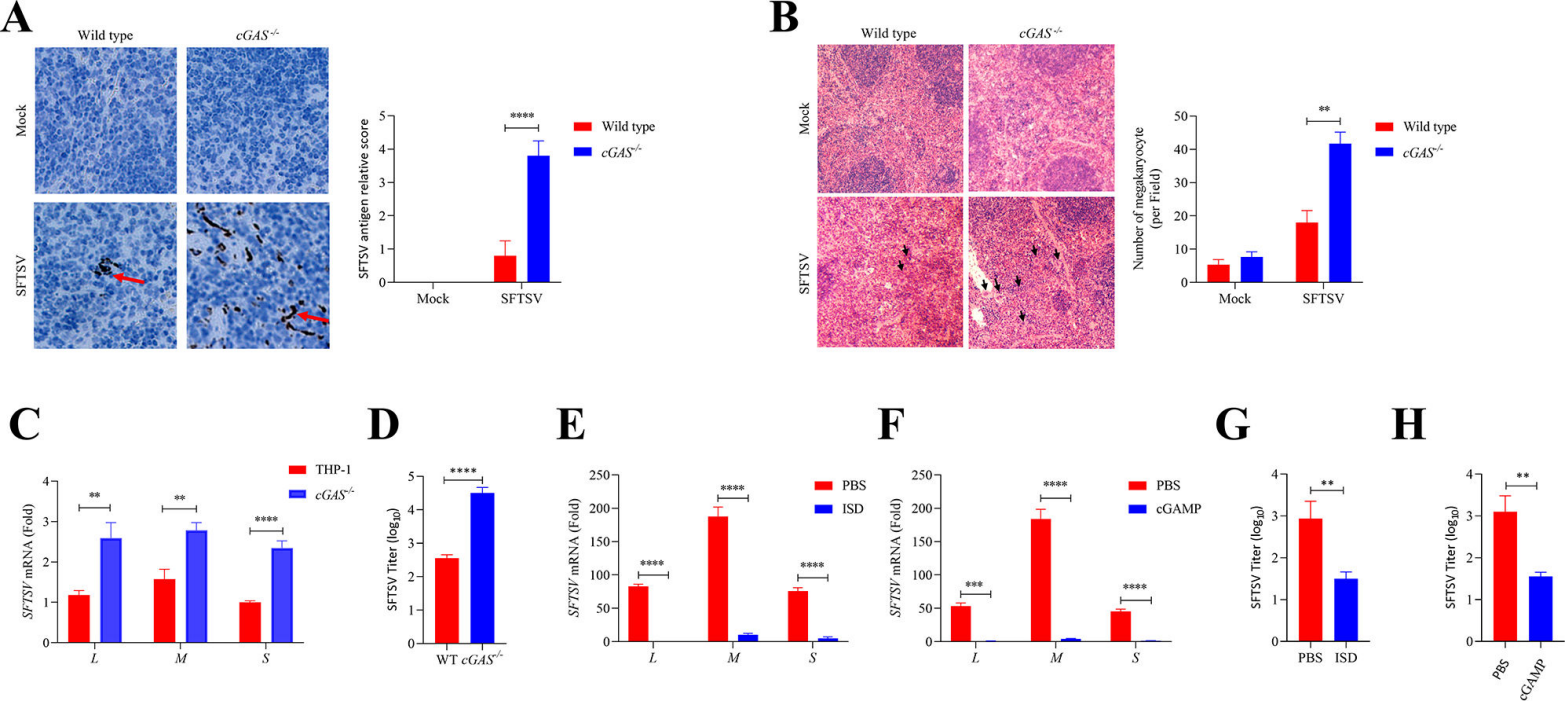
The impact of cGAS deficiency for host to suppress SFTSV replication *in vivo* and *in vitro*. (**A**) On day 3 post-SFTSV infection, the spleens of C57BL/6J wild-type and *cGAS^−/−^* mice were immunohistochemically stained. SFTSV-positive cells in the spleen of mice were shown in the figure. The detection of SFTSV was evaluated as the number of positive cells per section from each microscopic field through a ranked score of 0–4. The indications for the scores were as follows: 0, no positive cells; 1, 1–10 positive cells; 2, 11–30 positive cells; 3, 31–50 positive cells; 4, >50 positive cells (Student’s *t*-test). (**B**) Megakaryocytes in the H&E-stained spleen of C57BL/6J wild-type and *cGAS^−/−^* mice on day 3 post-SFTSV infection (Student’s *t*-test). (**C**) *cGAS* knockout and WT THP-1 cells were infected with SFTSV at MOI of 0 or 0.5 for 24 h. The transcriptional levels of SFTSV *L*, *M*, and *S* were detected with RT-qPCR (Student’s *t*-test). (**D**) *cGAS* knockout and WT THP-1 cells were infected with SFTSV at MOI of 0.5 for 24 h. Viral titer was measured with median tissue culture infective dose (TCID_50_) (Student’s *t*-test). (**E and F**) THP-1 cells were pretreated with interferon stimulatory DNA (ISD) (2 µg/mL) or cGAMP (2.5 µg/mL) for 6 h, and then, THP-1 cells were infected with SFTSV at MOI of 0.5 for 24 h. The transcriptional levels of SFTSV *L*, *M*, and *S* were detected with RT-qPCR (Student’s *t*-test). (**G and H**) THP-1 cells were pretreated with ISD (2 µg/mL) or cGAMP (2.5 µg/mL) for 6 h, and then, THP-1 cells were infected with SFTSV at MOI of 0.5 for 24 h. The culture supernatant was collected to detect viral titer with TCID_50_ (Student’s *t*-test).

As a conserved DNA PRR, cGAS is important in restricting DNA viruses and intracellular bacteria for maintaining host homeostasis (28, 34, 35). However, it is not clear whether DNA PRR cGAS plays a role in innate immunity against RNA virus SFTSV infection. To elaborate on the protective role of cGAS in SFTSV infection *in vivo*, C57BL/6J wild-type and *cGAS^−/−^* mice were challenged with SFTSV intraperitoneally to determine the difference of SFTSV infection between wild-type and *cGAS^−/−^* mice. We observed that the wild-type and *cGAS^−/−^* mice were both resistant to the infection of SFTSV (Fig. S2A), and no mouse died of SFTSV infection in both groups. Immunohistochemistry and hematoxylin and eosin (H&E) staining of mouse spleens were performed to evaluate the proliferation of SFTSV in wild-type and *cGAS^−/−^* mice. Increased SFTSV replication (Fig. 2A) and megakaryocytes (Fig. 2B) were observed in *cGAS^−/−^* mice, which indicated that *cGAS^−/−^* mice mounted worse immunity to SFTSV infection than wild-type mice. Additionally, RT-qPCR showed that infection of SFTSV in *cGAS^−/−^* mice led to decreased transcription of inflammatory cytokines and type I IFN but increased SFTSV replication compared to the wild-type mice (Fig. S2B). These results demonstrate that although cGAS deficiency is not lethal to SFTSV-infected mice, cGAS deficiency makes mice more vulnerable to SFTSV infection.

Furthermore, THP-1 cells were used to validate these findings *in vitro*. We observed higher transcriptional levels of the *L*, *M*, and *S* segments of SFTSV in *cGAS* knockout THP-1 cells (Fig. 2C) than wild-type cells. In addition, we further detected the SFTSV titers in the cell culture supernatant with TCID_50_. Compared to the wild type, *cGAS^−/−^*, THP-1 cells partially lost their ability to suppress the replication of SFTSV (Fig. 2D), indicating that SFTSV became more rampant in *cGAS* knockout cells. Since cGAS deficiency promotes SFTSV proliferation, conversely, cGAS activation should be able to inhibit SFTSV proliferation. ISD and cGAMP are effective stimulators for cGAS and STING, respectively. Subsequently, THP-1 cells were pretreated with ISD or cGAMP before SFTSV infection, and we observed that ISD or cGAMP pretreatment was effective in inhibiting SFTSV gene replication (Fig. 2E and F). In addition, the TCID_50_ assay showed that the amount of mature virion in the supernatant of THP-1 was significantly decreased under the stimulation of ISD or cGAMP (Fig. 2G and H). Together, these results indicate that cGAS is important for innate immunity against RNA virus SFTSV infection.

### cGAS recognizes RNA virus SFTSV infection via relocated mitochondrial DNA

**Fig 3:**
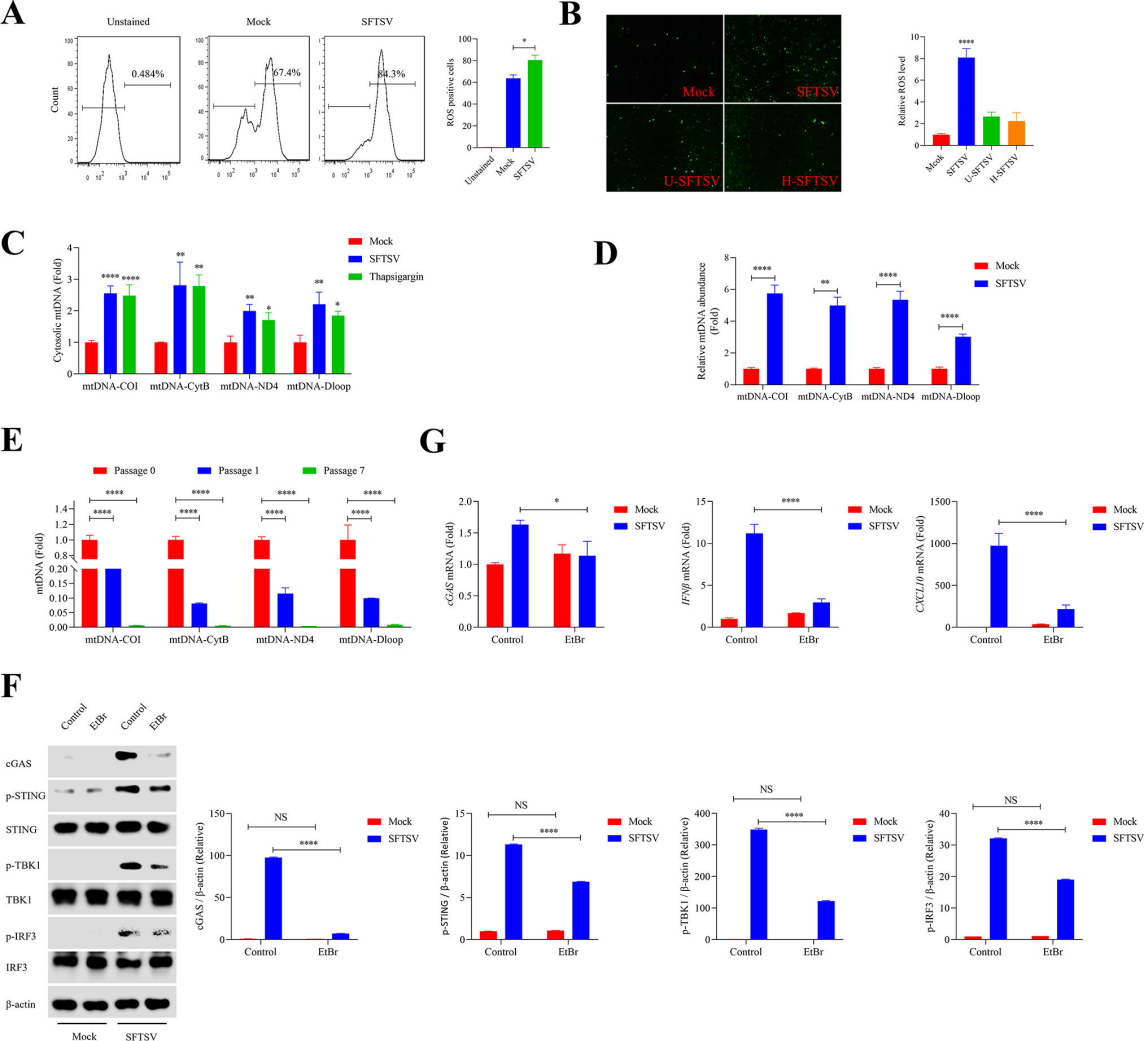
SFTSV infection induces the release of mtDNA. (**A**) THP-1 cells were infected with SFTSV at MOI of 0.5 for 24 h. The percentage of ROS-positive cells was detected by flow cytometry (one-way ANOVA). (**B**) A dichlorofluorescein diacetate fluorescence probe assay was used to detect the intracellular ROS level in HeLa cells (one-way ANOVA). (**C**) THP-1 cells were infected with SFTSV at MOI of 0.5 for 24 h or treated with thapsigargin (4 µg/mL) for 4 h. Cytosolic mtDNA sequences of COI, CytB, ND4, and Dloop were determined with qPCR (one-way ANOVA). (**D**) The levels of mitochondria-specific DNA sequences COI, CytB, ND4, and Dloop presenting in cGAS pulldown from SFTSV-infected THP-1 cells or control cells were tested with qPCR (Student’s *t*-test). (**E**) THP-1 cells were depleted of mtDNA with EtBr. Cytosolic mtDNA sequences of COI, CytB, ND4, and Dloop were detected with qPCR (one-way ANOVA). (**F and G**) THP-1 cells were cultured with EtBr for seven passages to deplete mtDNA and then infected with SFTSV at 0.5 MOI for 24 h. cGAS-related proteins were detected using immunoblot analysis. EtBr-untreated and treated THP-1 cells were infected with SFTSV at MOI of 0 or 0.5 for 24 h. The expression levels of cGAS, p-STING, p-TBK1, and p-IRF3 were semi-quantified with ImageJ software (two-way ANOVA). The transcriptional levels of *cGAS*, *IFNβ*, and *CXCL10* were detected with RT-qPCR (two-way ANOVA).

Mitochondrial DNA (mtDNA) is a well-known mitochondrial damage-associated molecular pattern (DAMP), which is considered as a classic stimulator of the antiviral cGAS-STING signaling pathway (36). To date, several studies have shown that cGAS could sense RNA viruses indirectly via mtDNA, such as Dengue virus and SARS-CoV-2 (31, 37). To determine whether mtDNA was involved in the activation of cGAS under SFTSV infection, cellular mitochondrial reactive oxygen species (ROS) was assayed via flow cytometry and fluorescence microscopy, which was tightly related to mitochondrial dysfunction (38). As expected, we observed that mitochondrial ROS production was significantly increased in SFTSV-infected cells (Fig. 3A and B). To further explore and characterize the role of mtDNA in the activation of the cGAS-STING signaling pathway in SFTSV-infected cells, cytosolic mtDNA was measured via qPCR, and thapsigargin was utilized as a positive control to induce mtDNA release. The results showed that SFTSV infection led to increased cytosolic mtDNA (Fig. 3C). Subsequently, qPCR was used to quantify the binding affinity of cGAS to cytosolic mtDNA under SFTSV infection. Mock and SFTSV-infected THP-1 cells were then collected, and the lysates were subjected to cGAS pulldown using anti-cGAS-specific antibodies. The DNA bound to cGAS was extracted, purified, and quantified with qPCR. Significant differences in the abundance of mtDNA fragments bound to cGAS were observed between mock THP-1 cells and SFTSV-infected THP-1 cells (Fig. 3D), which suggested that mtDNA was enriched and bound to cGAS under SFTSV infection. To further test the function of mtDNA in stimulating the cGAS pathway during SFTSV infection, ethidium bromide (EtBr) was used to deplete mtDNA (39), which reduced mtDNA by approximately 99% at the seventh passage of THP-1 cells (Fig. 3E). mtDNA depletion not only decreased the protein levels of cGAS, p-STING, p-TBK1, and p-IRF3 under SFTSV infection (Fig. 3F) but also blocked the activation of interferon response (Fig. 3G). Collectively, these results reveal that ectopic mtDNA is the ligand of cGAS in SFTSV-infected cells.

### The SFTSV N interacts with the 161-382 domain of cGAS

**Fig 4:**
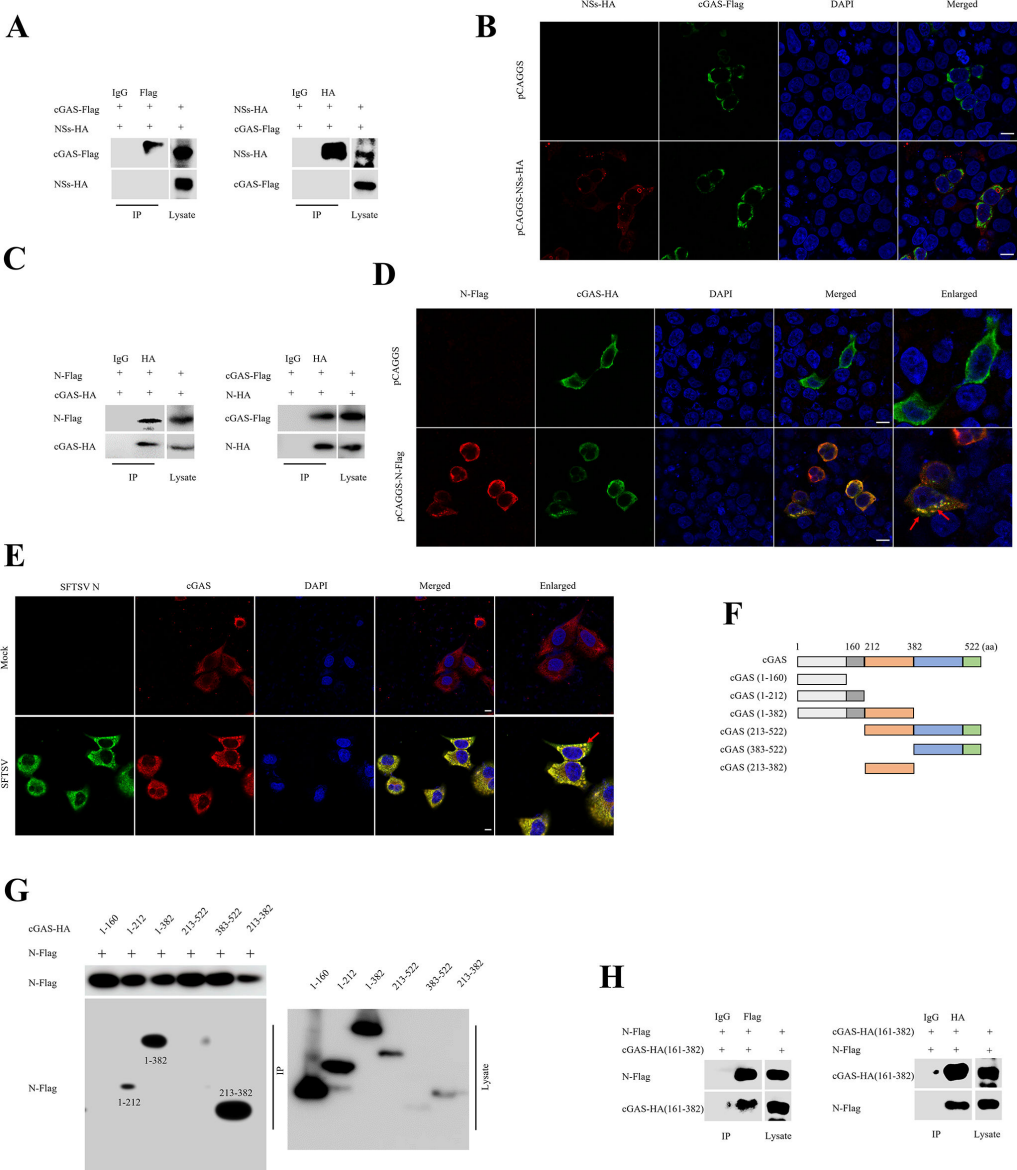
SFTSV N interacts with DNA sensor cGAS. (**A**) HEK293T cells were co-transfected with pCAGGS-NSs-HA and pCAGGS-cGAS-Flag for 24 h. Cell lysates were subjected to IP, and eluted proteins were detected using immunoblot analysis. (**B**) HEK293T cells were co-transfected with pCAGGS-NSs-HA and pCAGGS-cGAS-Flag for 24 h. Co-localization of NSs (red) and cGAS (green) was detected using confocal microscopy. Nuclei were stained with 4′,6-diamidino-2-phenylindole (DAPI) (blue). Scale bar, 10 µm. (**C**) HEK293T cells were co-transfected with pCAGGS-N-Flag and pCAGGS-cGAS-HA or pCAGGS-N-HA and pCAGGS-cGAS-Flag for 24 h. Cell lysates were subjected to IP, and eluted proteins were detected using immunoblot analysis. (**D**) HEK293T cells were co-transfected with pCAGGS-N-Flag and pCAGGS-cGAS-HA for 24 h. Co-localization of N (red) and cGAS (green) was analyzed with confocal microscopy. Nuclei were stained with DAPI (blue). (**E**) HeLa cells were infected with SFTSV at MOI of 0.5 for 24 h. Co-localization of SFTSV N (green) and cGAS (red) was analyzed using confocal microscopy. Nuclei were stained with DAPI (blue). (**F and G**) HEK293T cells were co-transfected with pCAGGS-N-Flag and cGAS truncations. Cell lysates were subjected to IP, and eluted proteins were detected using immunoblot analysis. (**H**) HEK293T cells were co-transfected with pCAGGS-N-Flag and pCAGGS-cGAS-HA (161-382). Cell lysates were subjected to IP, and eluted proteins were analyzed with immunoblot analysis.

It has been established that SFTSV NSs functions as an important viral virulence factor that hijacks antiviral innate immune molecules into IBs for immune escape (16, 18, 21). To explore whether NSs could capture cGAS, the interaction between NSs and cGAS was detected under NSs transient transfection status. Unfortunately, the co-immunoprecipitation (co-IP) assay showed that cGAS and NSs did not interact with each other (Fig. 4A), and confocal microscopy further confirmed that NSs exhibited different distribution patterns with cGAS (Fig. 4B). To further explore the components of SFTSV that could interact with cGAS, SFTSV N, the most abundant protein of SFTSV, was co-transfected with cGAS into HEK293T cells. To our surprise, the co-IP assay showed that N was pulled down by cGAS, and vice versa, which indicates that N interacted with cGAS (Fig. 4C). Moreover, confocal microscopy was performed to detect the co-localization between cGAS and N under co-transfection status in HEK293T cells. The results showed that N co-localized with cGAS (Fig. 4D). To further evaluate the mechanism of cGAS and N interaction, HeLa cells were utilized to detect the interaction of endogenous cGAS and SFTSV N under SFTSV infection. Similarly, confocal microscopy showed that there was a consistent distribution pattern of cGAS and N (Fig. 4E). To further evaluate the protein domain of cGAS captured by N, we mapped regions of cGAS and seven cGAS truncations, which were constructed based on the functional domains (Fig. 4F). Co-IP assays showed that the 161-382 domain of cGAS co-precipitated with N, indicating that this region mediated the interaction of cGAS and N (Fig. 4G). In addition, the co-IP assay showed that cGAS truncation (161-382 domain) could interact with N (Fig. 4H). Taken together, these data demonstrate that N binds to cGAS, which may connect with the immune escape of SFTSV.

### The SFTSV N inhibits the activation of cGAS-STING signaling pathway

**Fig 5:**
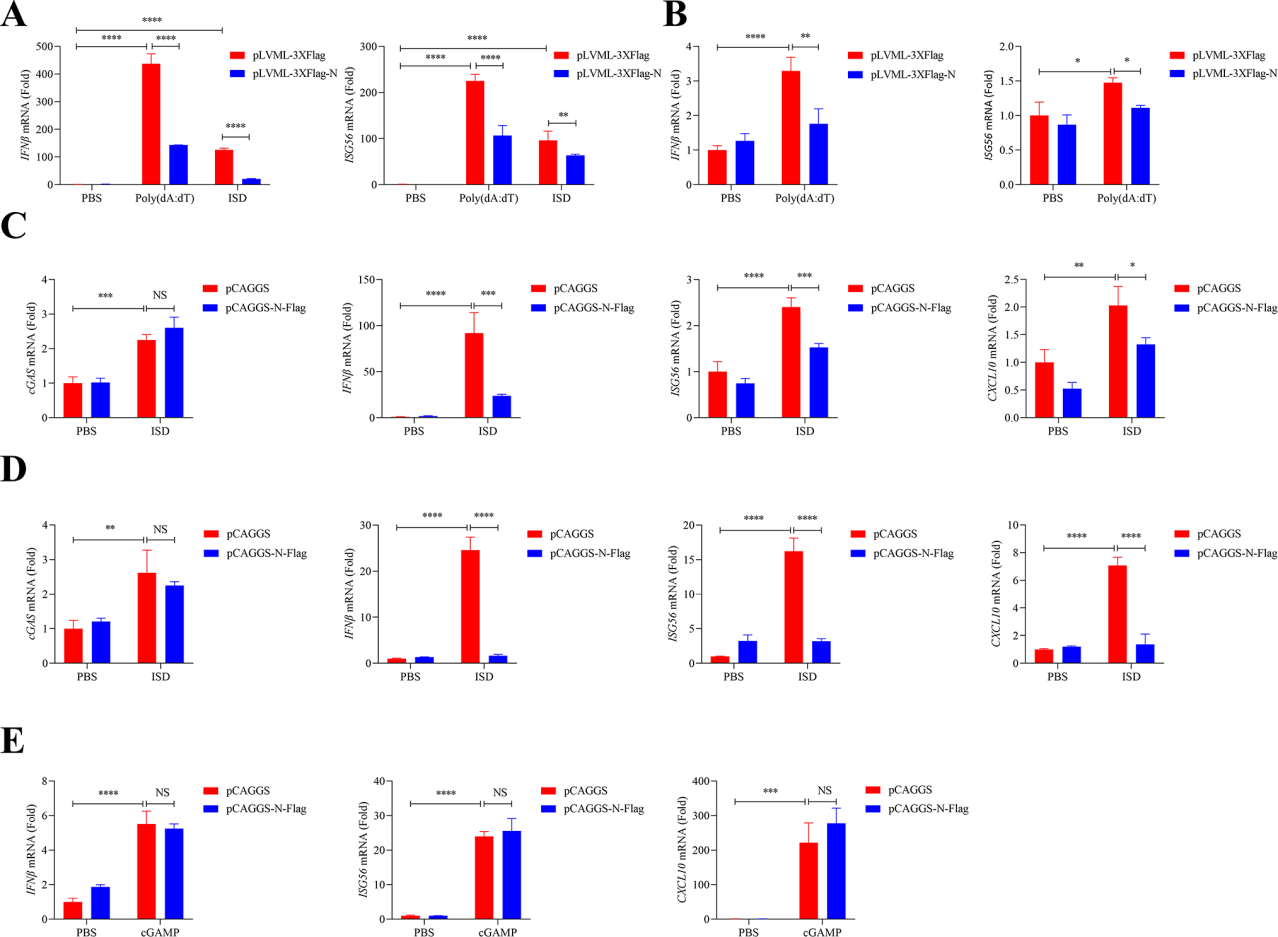
SFTSV N counteracts the cGAS-STING signaling pathway. (**A**) THP-1 cells were stably transfected with pLVML-3×Flag or pLVML-3×Flag-N followed by poly(dA:dT) (0.2 µg/µL) or ISD (2 µg/mL) treatment for an additional 6 h. The transcriptional levels of *IFNβ* and *ISG56* were determined with RT-qPCR (two-way ANOVA). (**B**) HeLa cells were stably transfected with pLVML-3×Flag or pLVML-3×Flag-N followed by poly(dA:dT) (0.2 µg/µL) treatment for an additional 6 h. The transcriptional levels of *IFNβ* and *ISG56* were determined with RT-qPCR (two-way ANOVA). (**C and D**) THP-1 cells or RAW cells were transiently transfected with pCAGGS-N-Flag or pCAGGS for 24 h, and then, ISD or phosphate buffered saline (PBS) were used to treat cells for 6 h, respectively. The transcriptional levels of *cGAS*, *IFNβ*, *ISG56*, and *CXCL10* were assayed with RT-qPCR (two-way ANOVA). (**E**) THP-1 cells were transiently transfected with pCAGGS-N-Flag or pCAGGS for 24 h, and then, cGAMP or PBS were used to treat cells for 6 h. The transcriptional levels of *IFNβ*, *ISG56*, and *CXCL10* were determined with RT-qPCR (two-way ANOVA).

Many viruses have evolved mechanisms to produce more viral progeny before the antiviral state is established in host cells. The cGAS-STING signaling pathway is a critical defense mechanism for the innate immune system, and our results have demonstrated that cGAS was effective in suppressing SFTSV infection (Fig. 2C and D). Therefore, it is worth exploring whether SFTSV has evolved the capability to suppress cGAS recognition. According to current reports, the strategies employed by RNA viruses to evade cGAS recognition included degradation, cleavage, or competitive inhibition (37, 40–42). Considering SFTSV N interacting with cGAS, we then investigated whether N could antagonize the cGAS-STING-mediated antiviral innate immune responses. Therefore, N stably expressing THP-1 and HeLa cells were constructed to examine whether N could inhibit the activation of the cGAS-STING signaling pathway in these cells. Poly(dA:dT) and ISD, stimulators of cGAS, were utilized as positive control to induce cGAS upregulation in N transfected THP-1 and HeLa cells. We found that both poly(dA:dT) and ISD induced a high expression of *IFNβ* and *ISG56* in THP-1 and HeLa cells, while the transcriptional levels of *IFNβ* and *ISG56* were significantly reduced in the overexpression of N (Fig. 5A and B). Subsequently, N transiently transfected THP-1 cells were treated with ISD to verify the inhibitory effect of N on cGAS. We found that overexpressing the N in THP-1 cells significantly inhibited the hyperexpression of *IFNβ*, *ISG56*, and *CXCL10* induced by ISD (Fig. 5C). In addition, mouse monocytic cells RAW264.7 were also utilized to evaluate the function of N under ISD stimulation. Unsurprisingly, the results showed that the gene expression of *IFNβ*, *ISG56*, and *CXCL10* was inhibited significantly in N overexpressing cells (Fig. 5D). STING is a downstream receptor protein of cGAS, which recognizes the signal molecule cGAMP and, thus, activates antiviral innate immune responses (28). To further confirm the block site of N on the cGAS-STING signaling pathway, the STING activator cGAMP was utilized to evaluate whether the expression of N could inhibit STING-mediated immune responses. The results showed that overexpressing N in THP-1 cells did not attenuate the transcriptional levels of *IFNβ*, *ISG56*, and *CXCL10* induced by cGAMP, which indicated that N did not block the activation of STING to suppress cGAS-STING-mediated antiviral innate immune responses (Fig. 5E). Collectively, SFTSV N is an important virulence factor to inhibit the cGAS-STING signaling pathway through interacting with cGAS.

### N induces the interaction of cGAS and LC3 to promote autophagy and degrade cGAS

**Fig 6:**
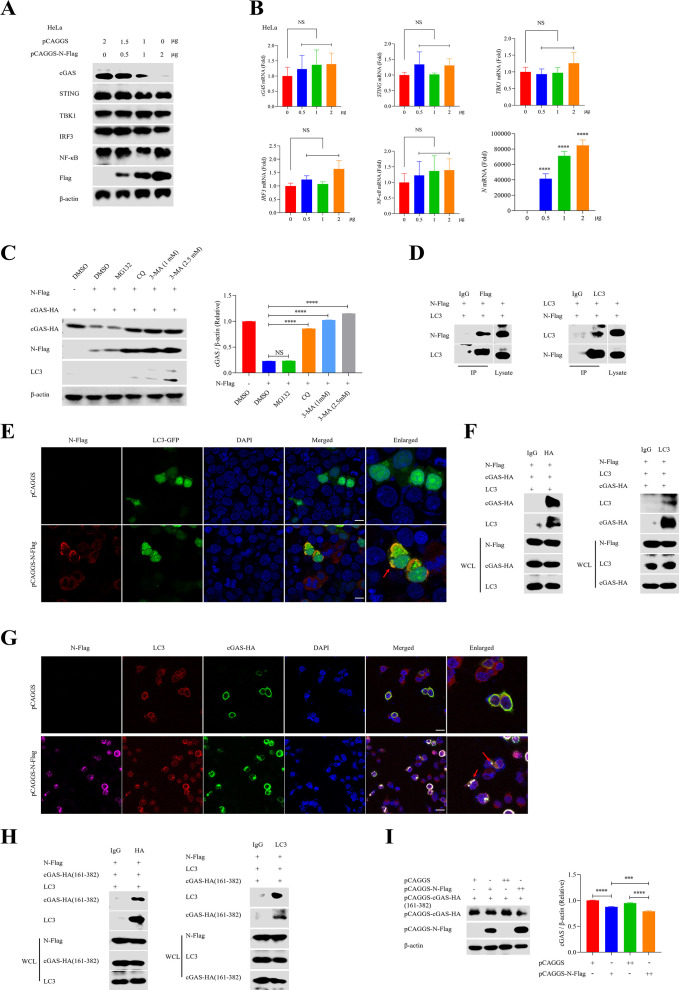
N induced the degradation of cGAS in an autophagy-dependent mechanism. (**A and B**) Different doses of pCAGGS-N-Flag were transfected into HeLa cells, and the deficient amount of plasmid DNA for each dose was reinforced with pCAGGS. cGAS-related proteins, including cGAS, STING, TBK1, IRF3, NF-κB, were analyzed with immunoblot. The transcriptional levels of *cGAS*, *STING*, *TBK1*, *IRF3*, and *NF-κB* were analyzed with RT-qPCR (one-way ANOVA). (**C**) Co-expression of pCAGGS-N-Flag (3 µg) and pCAGGS-cGAS-HA (2 µg) in HEK293T cells in the presence of dimethyl sulfoxide (DMSO), MG-132 (5 µM), chloroquine (CQ) (5 µM), or 3-MA (1 and 2.5 mM). cGAS-HA was assayed using immunoblot analysis. The gray values of cGAS-HA bands were semi-quantified with ImageJ (one-way ANOVA). (**D and E**) HEK293T cells were co-transfected with pCAGGS-N-Flag and LC3-GFP for 24 h. Cell lysates were subjected to IP, and eluted proteins were analyzed using immunoblot analysis. Co-localization of LC3-GFP (green) and N-Flag (red) was detected using confocal microscopy. Nuclei were stained with DAPI (blue). (**F**) HEK293T cells were co-transfected with pCAGGS-N-Flag, pCAGGS-cGAS-HA, and LC3-GFP for 24 h. Cell lysates were subjected to IP, and eluted proteins were detected using immunoblot analysis. (**G**) HEK293T cells were co-transfected with pCAGGS-cGAS-HA and pCAGGS-N-Flag for 24 h. Co-localization of endogenous LC3 (red), cGAS-HA (green), and N (purple) was detected using confocal microscopy. Nuclei were stained with DAPI (blue). (**H**) HEK293T cells were co-transfected with pCAGGS-N-Flag and pCAGGS-cGAS-HA (161-382). Cell lysates were subjected to IP, and eluted proteins were detected using immunoblot analysis. (**I**) HEK293T cells were co-transfected with pCAGGS-N-Flag and pCAGGS-cGAS-HA (161-382). The protein expression levels of cGAS truncation (161-382) and N-Flag were detected using immunoblot analysis. The gray values of cGAS truncation bands were semi-quantified with ImageJ (Student’s *t*-test).

To further explore the mechanism of N-mediated immunosuppression of the cGAS pathway, HeLa cells were utilized to investigate whether N could regulate the cGAS-STING signaling pathway. Immunoblot analysis showed that the overexpression of N decreased the protein levels of both endogenous and exogenous cGAS significantly in a dose-dependent manner (Fig. 6A; Fig. S3A). In addition, stable N expression in THP-1 and HeLa cells also led to the reduction of cGAS (Fig. S3B and C). However, the protein levels of STING, TBK1, IRF3, and NF-κB were not affected by the overexpression of N (Fig. 6A), which indicated that N might specifically target cGAS to inhibit the activation of the cGAS-STING signaling pathway. Moreover, we also found that the Ns of Rift Valley fever virus (RVFV) and Heartland bandavirus (HRTV), which are genetically close to SFTSV, also decreased cGAS protein levels in a dose-dependent manner (Fig. S3D). Furthermore, RT-qPCR results showed that the overexpression of N did not disturb the gene expression of *cGAS*, *STING*, *TBK1*, *IRF3*, and *NF-κB* (Fig. 6B; Fig. S3B and C). Collectively, these data demonstrate that SFTSV and other Bunyavirus N could inhibit the cGAS-STING signaling pathway through decreasing the protein level of cGAS.

Generally, proteasome and autophagy are two main pathways for protein degradation. Therefore, to further elucidate the mechanism of SFTSV N degradation of cGAS, proteasome and autophagy inhibitors were used to determine whether they could block N-induced cGAS reduction. Immunoblot analysis showed that treatment of autophagy inhibitor CQ and 3-MA could reverse N-induced cGAS reduction, while the proteasome inhibitor (MG132) did not (Fig. 6C). Beclin 1 plays a central role in autophagy, which involves the formation of autophagosomes to their extension and maturation (43). Therefore, N was transiently transfected in *Beclin 1* knockout HeLa cells to determine the protein level of cGAS. We found that the protein level of cGAS did not change significantly in response to N overexpression in *Beclin 1^−/−^* HeLa cells (Fig. S3E). Taken together, these results demonstrate that N promotes cGAS degradation in an autophagy-dependent manner.

To further explore the mechanism of N-induced cGAS degradation, LC3, an important autophagy marker, was co-expressed with N in HEK293T cells. The co-IP assay showed that LC3 could physically interact with N, and vice versa (Fig. 6D). Confocal microscopy also demonstrated the co-localization of exogenous LC3-GFP and N in HEK293T cells (Fig. 6E). In consideration of the interaction between N and LC3, we further speculated that N-induced cGAS degradation might be achieved by autophagy. Therefore, co-IP was utilized to determine whether N overexpression could promote the interaction between LC3 and cGAS. The co-IP assay showed that cGAS could interact with LC3 in the presence of N (Fig. 6F); the absence of N did not induce cGAS to interact with LC3 (Fig. S3F). In addition, confocal microscopy also showed that SFTSV N could induce the co-localization of cGAS and LC3 (Fig. 6G; Fig. S3G). Furthermore, the co-IP assay showed that N promoted the physical interaction of the truncation of cGAS (161-382 domain) with LC3 and induced cGAS truncation degradation in a dose-dependent manner (Fig. 6H and I; Fig. S3H). Taken together, these results demonstrate that SFTSV N mediates the interaction of cGAS and LC3, thereby promoting the degradation of cGAS through autophagy.

## DISCUSSION

SFTSV is a widespread tick-borne Bunyavirus in Asia causing a high case fatality rate of up to 30%. Currently, there are no effective drugs to treat SFTSV infection. Innate immunity is the first line of defense to combat invading pathogens, which uses PRRs to recognize DAMPs and PAMPs to activate effective immune responses. To date, multiple RNA PRRs, such as RIG-I, MDA5, and SAFA, have been reported to recognize the genomic RNA of SFTSV to establish antiviral innate immune responses (18, 26). Here, we further found that the expression levels of DNA sensor cGAS were increased significantly during SFTSV infection, and the upregulation of cGAS could significantly inhibit SFTSV replication. These results suggested that DNA sensor cGAS played a role in recognizing and restricting RNA virus SFTSV infection. cGAS, a DNA sensor, can detect pathogenic DNA to trigger type I interferon response against DNA virus or microbial organism (28). Recent studies showed that cGAS also has a striking role against +ssRNA viruses (30, 31, 37, 42). The mechanism of cGAS against RNA viruses is through recognizing DNA released from mitochondria (30, 31, 37, 42) or extranuclear genomic DNA (32). On this basis, we found that −ssRNA SFTSV infection stimulated mtDNA release into the cytoplasm, and the depletion of mtDNA could block the activation of cGAS-mediated immune responses. These results demonstrated that relocated mtDNA was the target of cGAS to recognize SFTSV. The activation of BAK/BAX has been demonstrated to be responsible for mtDNA release under SFTSV infection (44). In addition, SFTSV infection led to the increased expression of a number of inflammatory cytokines, such as IL-1β and TNF (45). Recent reports indicated that IL-1β and TNF could trigger the release of mtDNA to activate the cGAS-STING signaling pathway (46, 47); thus, increased IL-1β and TNF might also contribute to the enrichment of mtDNA in the cytoplasm.

We have also found that SFTSV has developed sophisticated tactics to suppress cGAS-mediated antiviral responses. Our study indicated that SFTSV N binds to cGAS and traps it into N-induced autophagy and eventually degrades in the autolysosomes, therefore suppressing cGAS-mediated antiviral immune responses. This degradation was rescued by treatment with autophagy inhibitors. We also observed a similar phenomenon on the N of Bunyaviruses RVFV and HRTV, which are genetically close to SFTSV. However, although autophagy was activated during SFTSV infection via N, it seemed not enough to reverse the massive expression of cGAS caused by released mtDNA and positive feedback of the IFN pathway (48) during SFTSV infection. Moreover, although our results revealed that N could inhibit cGAS-mediated immune responses, there was no significant change in the mRNA level of cGAS. Our hypothesis is that under the stimulation of a large amount of exogenous DNA, a variety of cytokines produced in the cells have made the cGAS transcriptional levels peak, but the degradation of cGAS protein has blocked the cGAS-STING signaling pathway, so there are still differences in the transcriptional levels of interferon and inflammatory cytokines. It is worth noting that SFTSV N is the most abundant SFTSV polypeptide and typically considered as a viral capsid protein that mainly functions as the component of the viral ribonucleoprotein essential for SFTSV RNA replication, transcription, and synthesis (49, 50). Despite a recent study showing that SFTSV N mediated the activation of classic autophagy in a Beclin 1-dependent manner (51), based on our knowledge, limited studies have revealed the underlying role of N, as an indispensable part of the virion, in innate immunity. Here, our results not only expanded that SFTSV N could mediate the activation of autophagy directly in a LC3-dependent manner but also suggested that SFTSV N also functioned as an important SFTSV virulence factor that mediated the interaction of cGAS and LC3 and promoted the degradation of cGAS in an autophagy-dependent manner.

N is a sequence of highly conserved proteins in the order *Bunyavirales* (52–54). Previous studies showed that RVFV could utilize its N to induce autophagy and, therefore, dampen antiviral innate immune responses by N to promote its replication (55), indicating that Bunyavirus N could be conserved in viral immune escape via autophagy induction. Several studies have reported that N assumed various immunosuppressive roles in *Hantavirus*. Andes virus is the only *Hantavirus* known to spread from person to person, and N has been reported to be an important antiviral element of Andes virus to circumvent innate immunity through inhibiting TBK1 autophosphorylation (56). Besides, Andes virus N promoted the expression of viral proteins via inhibiting protein kinase R-induced translational shutdown (57). In addition, *Hantaan* virus N could downregulate the expression of *IFNβ* (58) and suppress the function of NF-κB via inhibiting NF-κB nuclear translocation (59). The ways of Andes virus and *Hantaan* virus in achieving immune escape further suggest that Bunyavirus N functions as an important virulence factor in mediating viral immune escape. We further indicated that Bunyavirus SFTSV, RVFV, and HRTV could degrade cGAS through N-induced autophagy to suppress cGAS-mediated antiviral immune responses. Taken together, these results suggest that N is a key molecule to perform multiple functions in Bunyavirus, but its ability to mediate immune escape has been neglected.

Current studies indicated that various RNA viruses, such as West Nile virus, influenza A virus, and SARS-CoV-2, are recognized by DNA sensor cGAS (29–32). It is mainly due to the invasion of RNA viruses that induce the release of mtDNA, which functions as a cGAS target. As countermeasures, RNA viruses have evolved different strategies to antagonize cGAS to evade the surveillance of host innate immunity. Dengue virus has been reported to block cGAS-mediated immune responses via viral protein NS2B, which targeted cGAS for autophagy-dependent degradation (37). A similar mechanism was also utilized by Chikungunya virus capsid protein (41). Besides, Zika virus NS1 promoted the cleavage of cGAS by caspase-1 (42). In addition, SARS-CoV-2 protein N competed with cGAS to bind to G3BP1, a co-factor of cGAS, to damage the abilities of cGAS to recognize DNA (40). Up to now, the studies on the immune escape of SFTSV mainly focus on NSs, which function to attack the innate immune system through sequestering key IFN-related molecules into IBs (16, 21, 22). However, we demonstrated that SFTSV NSs did not have the ability to trap cGAS into IBs to achieve the immune escape. Furthermore, a novel virulence factor, the N of SFTSV, showed its function of mediating immunosuppression through triggering autophagy to degrade cGAS. Furthermore, we uncovered that N promotes autophagy-dependent cGAS degradation via recognizing the 161-382 domain of cGAS. Although the truncation 212-382 of cGAS could interact with N, the interaction of N and 213-522 domain of cGAS is not observed. The result could be explained that the recognition site of N in the 213-382 domain of cGAS may be covered up by the 283-522 domain, and the underlying interaction site is worth further investigation.

**Fig 7:**
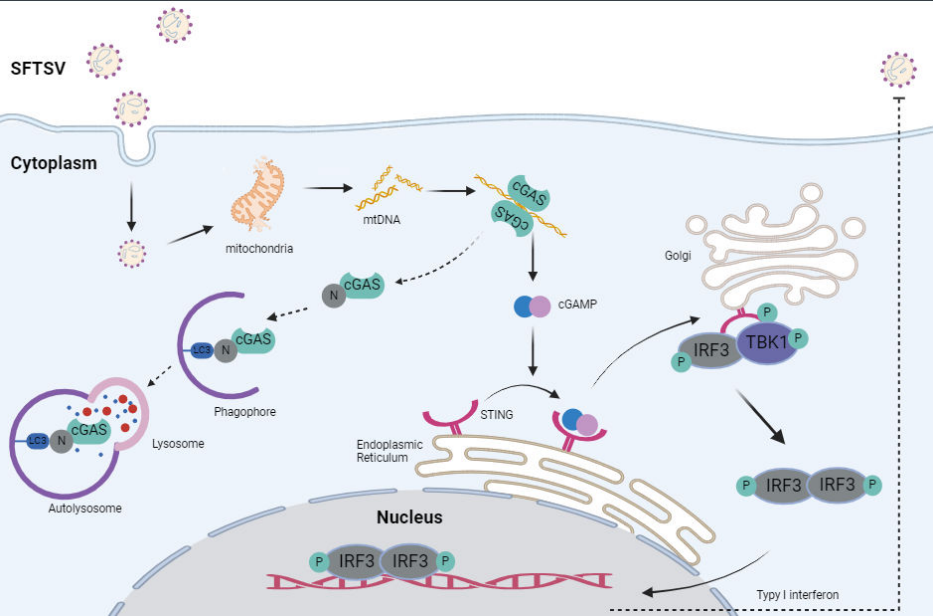
Schematic model illustrating the interaction between SFTSV and DNA sensor cGAS. SFTSV infection triggers the release of mtDNA to the cytoplasm; thus, cytosolic mtDNA binds to cGAS and activates the cGAS-STING signaling pathway. Type I interferons are manufactured to suppress SFTSV infection. In addition, cGAS and LC3 were linked together by SFTSV N and trapped in N-induced autophagy to degrade cGAS.

In this study, we systemically investigated the complicated interaction of SFTSV and the cGAS-STING signaling pathway. The cGAS-STING signaling pathway is responsible for sensing SFTSV infection via recognizing released mtDNA in the cytoplasm. More importantly, we find that SFTSV N suppresses cGAS-STING antiviral immune response by the degradation of cGAS in N-induced autophagy (Fig. 7). These findings expand our knowledge on SFTSV N as a novel virulence factor of SFTSV and its pathogenic mechanisms in suppressing host antiviral immune responses.

## MATERIALS AND METHODS

### Cell lines and viral infection

HEK293T, Vero, RAW264.7, and HeLa cells were cultured in Dulbecco's modified eagle medium (DMEM). *Beclin 1^−/−^* HeLa cells were kindly provided by Dr. Wen-Sheng Wei (Peking University, Beijing, China). THP-1 cells were cultured in RPMI 1640 medium. Phorbol 12-myristate 13-acetate (PMA) (100 ng/mL) was used to promote the differentiation of THP-1 monocytes into macrophages. Briefly, after 24-h treatments, PMA was removed from the medium, and THP-1 cells were cultured for an additional 24 h. SFTSV (strain JS2011-013-1) was used in this study. For viral infection, differentiated THP-1 cells were infected with SFTSV at the indicated multiplicity of infection (MOI = 0.5) for 2 h at 37°C.

### Mice

C57BL/6J *cGAS^−/−^* mice were purchased from Jackson Laboratories (Ellsworth, ME). All mice were of C57BL/6J genetic background, and all experiments were carried out with age- and gender-matched mice (6–8 weeks old, female). For the SFTSV infection model, mice were anesthetized using ketamine and xylazine (45 and 10 mg/kg) and then intraperitoneally incubated with 10 lethal doses of SFTSV. Mice were sacrificed on day 3 post-infection for sample collection. The animal study was approved by the ethics committee of Wuhan University (2019YF2013) and was conducted in accordance with the guidelines for the protection of animal subjects.

### Lentiviral vectors

The shRNA cGAS sequence is 5′-GCGTTAGAAACACTTCAAGAA-3′. Guide RNA sequences are CACCGCCCGGAGCTTCGAGGCCCCAGG. The sequence of SFTSV N was downloaded from NCBI (https://www.ncbi.nlm.nih.gov/). shRNA, lentiCRISPR v2, pLVML-3×Flag-N, and pLVML-3×Flag plasmids were co-transfected with the packaging plasmids psPAX2 and pMD2.G into HEK293T cells to generate lentivirus. psPAX2 and PMD2.G are the packing plasmid and envelope plasmid of lentivirus, respectively. psPAX2 (Addgene plasmid # 12260; http://n2t.net/addgene:12260; RRID:Addgene_12260) and pMD2.G (Addgene plasmid # 12259; http://n2t.net/addgene:12259; RRID:Addgene_12259) were gifts from Didier Trono. Lentiviral particles were used to infect cells in the presence of polybrene (8 µg/mL). Cells were selected with puromycin (1 µg/mL) for 10 days followed by 24-h lentivirus treatment.

### Co-immunoprecipitation and immunoblot assay

HEK293T cells were lysed with 1 mL NP-40 lysis buffer (20 mM Tris, pH 7.4, 150 mM NaCl, 1 mM EDTA, 1% Nonidet P-40, 10 µg/mL aprotinin) for 30 min on ice. Collected cell lysates were incubated with the indicated antibodies (0.5 µg) and protein G sepharose beads (25 µL) at 4°C for 4 h. The protein-bound beads were then collected and washed three times with lysis buffer (0.75 mL) containing 0.5 M NaCl. SDS loading buffer was added into the sample supernatants and then boiled for 10 min at 95°C. Samples were electrophoresed in SDS-PAGE gels and then transferred onto the polyvinylidene fluoride membrane and incubated with the antibody at 4°C overnight. After incubation with the HRP-conjugated secondary antibody for 1 h, the protein band was detected using the SuperSignal West Pico Chemiluminescent Substrate Kit (Thermo Scientific, Rockford, IL).

The antibodies used were as follows: anti-cGAS rabbit (15102S, Cell Signaling Technology), anti-STING rabbit (13647S, Cell Signaling Technology), anti-phospho-STING (Ser366) rabbit (19781S, Cell Signaling Technology), anti-TBK1 (Ser172) rabbit (3504S, Cell Signaling Technology), anti-phospho-TBK1 (Ser172) rabbit (5483S, Cell Signaling Technology), β-actin (sc-47778, Santa Cruz Biotechnology), anti-HA rabbit (51064-2-AP, Proteintech), anti-HA mouse (66006-2-Ig, Proteintech), anti-FLAG rabbit (20543-1-AP, Proteintech), anti-FLAG mouse (66008-3-Ig, Proteintech), anti-LC3 rabbit (ab48394, Abcam), anti-TOM20 rabbit (42406, Cell Signaling Technology), anti-TIM23 mouse (sc-514463, Santa Cruz Biotechnology), anti-OPTN rabbit (DF6655, affbiotech), anti-NDP52 rabbit (DF12360, affbiotech), and anti-COX2 rabbit (55070-1-AP, Proteintech).

### Production of anti-SFTSV-N antibody

Monoclonal anti-N was isolated using recombinant SFTSV N protein as a bait from a phage-display antibody library derived from the peripheral blood mononuclear cells of a patient who recovered from SFTS disease. The variable region of anti-N antibody fused with the human heavy chain constant region gene (IgG1) by overlapping PCR and then cloned into pCAGGS eukaryotic expression vector. The recombinant expression vectors were transfected into HEK293T cells; then, anti-N antibody was purified using a protein A column (GenScript, L00464) and a Superdex 200 column (Cytiva, 28,989,335).

### Immunofluorescence and confocal microscopy

Immunofluorescence assays were performed to study the subcellular localization of proteins. The treated HEK293T and HeLa cells were fixed with 4% paraformaldehyde for 15 min, permeabilized with 0.2% Triton X-100 for 20 min, and blocked with 5% bovine serum albumin for 30 min. The corresponding primary antibodies were incubated overnight at 4°C, and fluorescently labeled secondary antibodies were stained for 1 h. The DAPI was used to counterstain the nuclei. Cells were observed using the Olympus IX73 fluorescent inverted microscope for immunofluorescence and Leica SP8 confocal laser microscope with 63× objective for confocal microscopy. All image analyses were performed using the software Leica Application Suite X.

### RT-qPCR

RNA was extracted with TRIzol (Thermo Fisher, Shanghai, China) and reverse-transcribed to cDNA with the High-Capacity cDNA Reverse Transcription Kit (Thermo Fisher, Shanghai, China). qPCR was performed with ChamQ Blue Universal SYBR qPCR Master Mix (Vazyme, Nanjing, China) (Stage 1: 95°C for 30 s, Stage 2: 95°C for 10 s and 60°C for 30 s, 40 cycles). Relative mRNA concentrations were calculated by the 2^−ΔΔCt^ method, normalizing with β-actin. The primers used are listed in Table 1.

**TABLE 1:** Primers used for RT-qPCR

Genes	Forward	Reverse
*cGAS*	TGTGGATATAACCCTGGCTTTG	GCTTTAGTCGTAGTTGCTTCCT
*STING*	AGCATTACAACAACCTGCTACG	GTTGGGGTCAGCCATACTCAG
*TBK1*	GGATCACTGCCATTTAGACCC	CAGGCATGTCTCCACTCCAG
*IRF3*	CCATCGGCTTTTGGGTCTGT	CTCGAACTCCCACTCTTCCC
*NF-κB*	ATGTGGAGATCATTGAGCAGC	CCTGGTCCTGTGTAGCCATT
*IFNβ*	CATTACCTGAAGGCCAAGGA	CAATTGTCCAGTCCCAGAGG
*ISG56*	GCGCTGGGTATGCGATCTC	CAGCCTGCCTTAGGGGAAG
*CXCL10*	GTGGCATTCAAGGAGTACCTC	TGATGGCCTTCGATTCTGGATT
*TNFα*	CCAGGGACCTCTCTCTAATCA	TCAGCTTGAGGGTTTGCTAC
*IL-6*	ACTCACCTCTTCAGAACGAATTG	CCATCTTTGGAAGGTTCAGGTTG
*SFTSV L*	AGTCTAGGTCATCTGATCCGTTTAG	TGTAAGTTCGCCCTTTGTCCAT
*SFTSV M*	AAGAAGTGGCTGTTCATCATTATTG	GCCTTAAGGACATTGGTGAGTA
*SFTSV N*	TGTCAGAGTGGTCCAGGATT	ACCTGTCTCCTTCAGCTTCT
*Dloop*	CATCTGGTTCCTACTTCAGGG	CCGTGAGTGGTTAATAGGGTG
*COI*	AGGCTTCTCAAATCATGAAA	ATTCATCGGCGTAAATCTAA
*ND4*	CCTCGTAGTAACAGCCATTC	TTGAAGTCCTTGAGAGAGGA
*CytB*	CCATCCTCCATATATCCAAA	CCAATGATGGTAAAAGGGTA
*M IFNβ*	TCCGAGCAGAGATCTTCAGGAA	TGCAACCACCACTCATTCTGAG
*M ISG56*	GTCCGGTTAAATCCAGAAGATCC	GCTTTGTCTACGCGATGTTTCC
*M CXCL10*	CCAAGTGCTGCCGTCATTTTC	GGCTCGCAGGGATGATTTCAA

### Cytosolic mtDNA selection

THP-1 cell lysates were isolated with lysis buffer (150 mM NaCl, 50 mM HEPES, pH 7.4, and 20 µg/mL digitonin). Cells were incubated with the buffer for 10 min at 4°C using end-to-end rocking. The supernatant was then centrifuged at 1,000 × *g* for 10 min and 20,000 × *g* for 20 min. DNA was subsequently extracted from the resulting supernatant with the QIAamp DNA Mini Kit (Qiagen). qPCR analysis was performed to measure mitochondrial DNA.

### Mitochondrial DNA depletion

THP-1 cells were treated with EtBr (100 ng/mL), sodium pyruvate (100 µg/mL), and uracil (50 µg/mL) for seven passages; then, cells were cultured with RPMI 1640 in the absence of EtBr for 1 day. Cytosolic mtDNA was detected with qPCR.

### Intracellular ROS detection

Intracellular ROS was detected using a dichlorofluorescein diacetate fluorescence probe assay with a Reactive Oxygen Species Assay Kit (Beyotime, Shanghai, China). The results were observed under an inversion fluorescence microscope.

### Enzyme-linked immunosorbent assay

Quantification of cGAMP was analyzed using the 2′3′-cGAMP ELISA Kit (Cayman).

### Flow cytometry

To measure mitochondrial ROS, cells were stained with 2,4-dinitrophenylhydrazine solution from the FlowCellect Oxidative Stress Characterization Kit (Thermo Fisher, Shanghai, China). Data were acquired with a BD FACSCanto II flow cytometer at a blue laser (488 nm) light source and analyzed with FlowJo.

### Immunohistochemistry

Tissues were fixed in 10% neutral buffered formalin for 36 h and transferred to 70% ethanol. Four-micrometer sections from the paraffin embedding tissues were stained with H&E. For immunohistochemistry (IHC) analysis, after deparaffinization and rehydration, 4-µm-thick sections were boiled in 10-mM citrate buffer and quenched endogenous peroxidase in 3% (vol/vol) H_2_O_2_, with the citrate buffer (10% target-retrieval solution) at 90°C for 30 min. An SFTSV-specific antibody was used for detecting SFTSV antigens by IHC.

### Statistical analysis

Data shown were performed at least three times and analyzed with GraphPad Prism Software by using Student’s *t*-test or one-way ANOVA. Error bars in the figures represent the mean ± SD, and *P* < 0.05 was considered statistically significant. ^*^P < 0.05, ^**^P < 0.001, ^***^P < 0.0001, and ^****^P < 0.00001; NS, not significant.

## Data Availability

This study includes no data deposited in external repositories.

## SUPPLEMENTAL MATERIAL

Supplemental material spectrum.03796-23-s0001.pdf

ASM does not own the copyrights to Supplemental Material that may be linked to, or accessed through, an article. The authors have granted ASM a non-exclusive, world-wide license to publish the Supplemental Material files. Please contact the corresponding author directly for reuse.
