# Supplementary material for: SFTSV nucleoprotein mediates DNA sensor cGAS degradation to suppress cGAS-dependent antiviral responses
Source: Microbiol Spectr. 2024 May 7;12(6):e03796-23. doi: 10.1128/spectrum.03796-23 (PMC11237745; doi:10.1128/spectrum.03796-23)
Supplement: Supplemental material [file spectrum.03796-23-s0001.pdf]

Supplementary Materials for

SFTSV nucleoprotein mediates DNA sensor cGAS degradation to suppress cGAS-dependent antiviral responses

Ze-zheng Jiang, Min Chu *et al.*

*Corresponding authors: Hui-Ju Han, hanhuiju@sdfmu.edu.cn;
Xue-jie Yu, yuxuejie@whu.edu.cn; Chuan-min Zhou, 00033465@whu.edu.cn;

This PDF file includes:

Figs. S1 to S3
Legends for Figs. S1 to S3

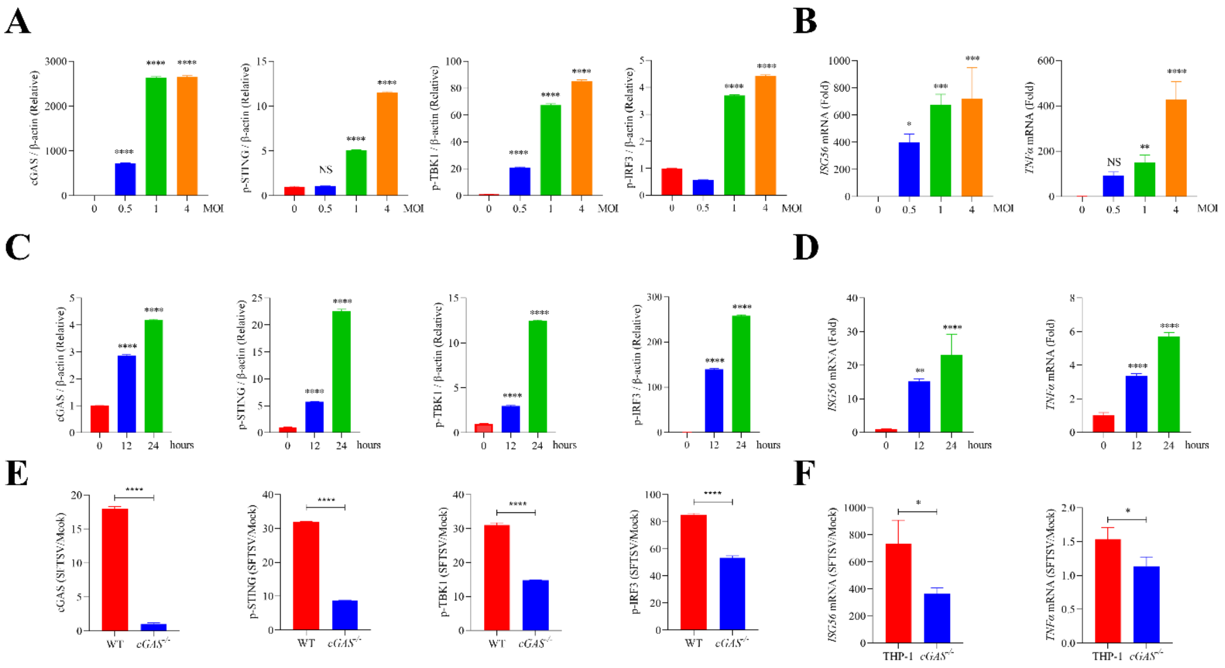

Fig. S1

SFTSV infection activates the intracellular DNA sensor cGAS. (A-B) THP-1 cells were infected with SFTSV at MOI of 0, 0.5, 1, and 4 for 24 h. The expression of cGAS, p-STING, p-TBK1, and p-IRF3 were semi-quantified with ImageJ software. The transcriptional levels of *ISG56*, *TNFα* were detected with RT-qPCR (one-way ANOVA). **(C-D)** THP-1 cells were infected with SFTSV at MOI of 0.5 for 0, 12, and 24 h. The expression levels of cGAS, p-STING, p-TBK1, and p-IRF3 were semi-quantified with ImageJ software. The transcriptional levels of *ISG56*, *TNFα* were assayed with RT-qPCR (one-way ANOVA). **(E-F)** WT and *cGAS* knockout THP-1 cells were infected with SFTSV at MOI of 0 or 4 for 24 h. The fold increases of cGAS, p-STING, p-TBK1, and p-IRF3 were semi-quantified with ImageJ software (Student's t test). The transcriptional levels of *ISG56*, *TNFα* were determined with RT-qPCR, the fold increases of mRNA (SFTSV/Mock) were compared by Student's t test.

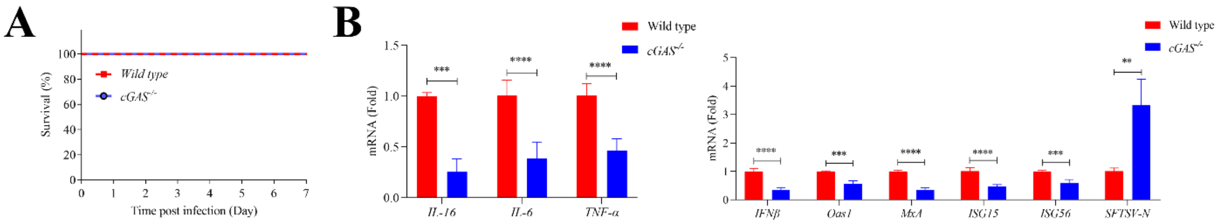

Fig. S2

cGAS is important for host to suppress SFTSV replication. (A) C57BL/6J wild type and *cGAS*$^{-/-}$ mice were challenged with 10 lethal doses of SFTSV by intraperitoneal injection. The lethal doses were detected using *IFNAR*$^{-/-}$ A129 mice. Survival of wild type and *cGAS*$^{-/-}$ were monitored for up to 16 days. Kaplan-Meier survival curves were obtained using GraphPad. **(B)** The transcription of *IL-1β*, *IL-6*, *TNFα*, and *IFNβ*, and relative ISGs including *Oas1*, *MxA*, *ISG15*, *ISG56*, and *N* were determined with RT-qPCR in the spleens of wild type and *cGAS*$^{-/-}$ mice (Student's t test).

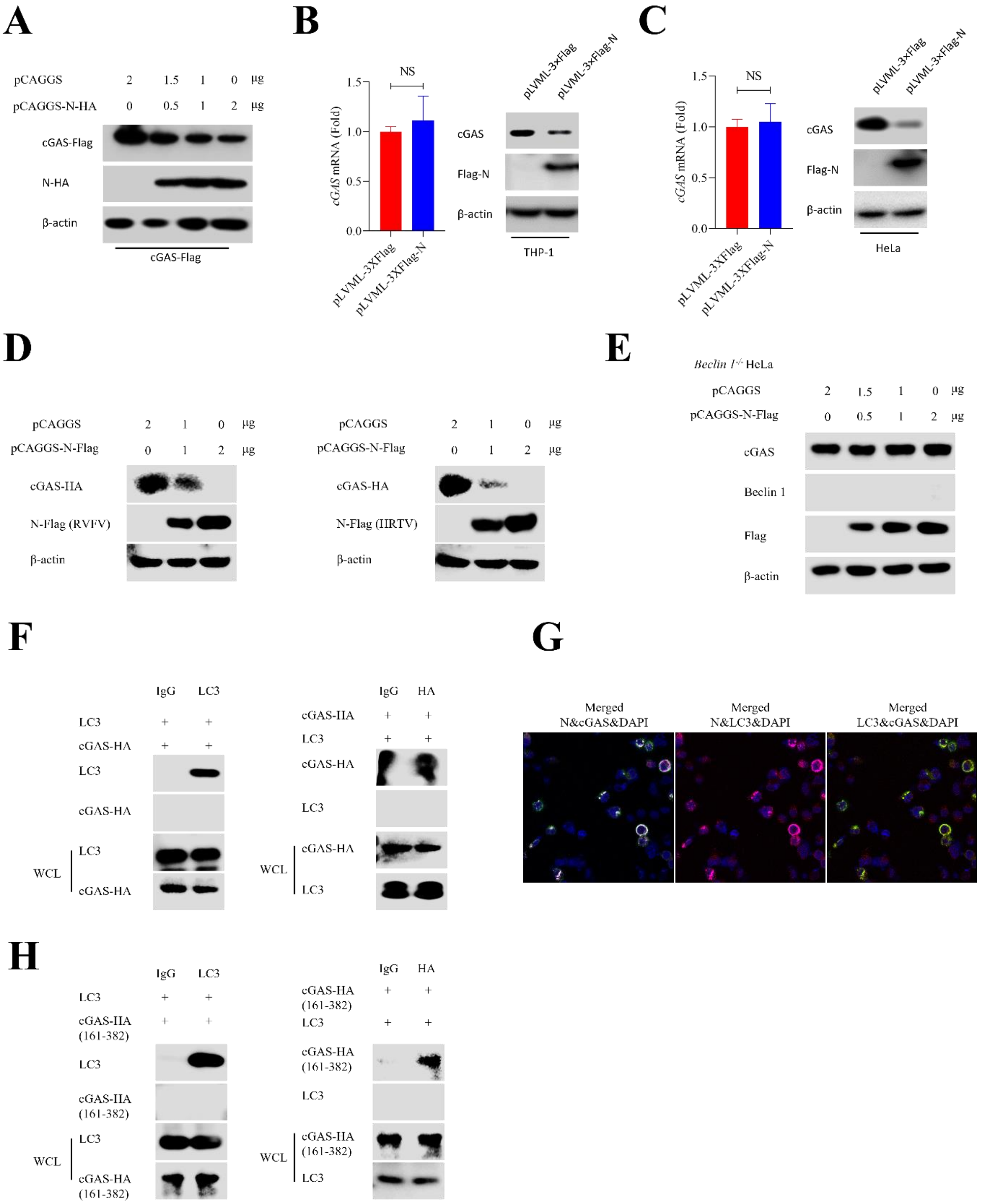

Fig.S3

N induced the degradation of cGAS in an autophagy-dependent mechanism. (A) HEK293T cells were co-transfected with pCAGGS-N-HA (dose-dependent manner) and pCAGGS-cGAS-Flag. cGAS was determined with immunoblot analysis. **(B-C)** THP-1 cells and HeLa cells were stably expressed with pLVML-3×Flag or pLVML-3×Flag-N. The proteins or transcriptional levels of cGAS were determined with immunoblot analysis or RT-qPCR (Student’s t test). **(D)** HEK293T cells were co-transfected with

pCAGGS-RVFV-N-Flag or pCAGGS-HRTV-N-Flag (dose-dependent manner) and pCAGGS-cGAS-HA. The protein levels of cGAS were determined with immunoblot analysis. **(E)** Different doses of pCAGGS-N-Flag were transfected into *Beclin 1*$^{-/-}$ HeLa cells and the deficient amount of plasmid DNA for each dose was reinforced with pCAGGS. The protein levels of cGAS were determined with immunoblot analysis. **(F)** HEK293T cells were co-transfected with pCAGGS-cGAS-HA and LC3-GFP for 24 h. Cell lysate were subjected to IP and eluted proteins were detected using immunoblot analysis. **(G)** A 2-way merge of Figure.6G for N and cGAS, N and LC3, LC3 and cGAS were provided. **(H)** HEK293T cells were co-transfected with pCAGGS-cGAS-HA (161-382) and LC3-GFP for 24 h. Cell lysate were subjected to IP and eluted proteins were detected using immunoblot analysis.
